# Exploration of the Topical Nanoemulgel Bearing with Ferulic Acid and Essential Oil for Diabetic Wound Healing

**DOI:** 10.3390/pathophysiology31040049

**Published:** 2024-11-25

**Authors:** Urati Anuradha, Valamla Bhavana, Padakanti Sandeep Chary, Nitin Pal Kalia, Neelesh Kumar Mehra

**Affiliations:** 1 Department of Biological Sciences, National Institute of Pharmaceutical Education and Research (NIPER), Hyderabad 500037, Telangana, India; uratianuradha23@gmail.com (U.A.); nitin.kalia@niperhyd.ac.in (N.P.K.); 2 Pharmaceutical Nanotechnology Research Laboratory, Department of Pharmaceutics, National Institute of Pharmaceutical Education and Research (NIPER), Ministry of Chemicals and Fertilizers, Government of India, Hyderabad 500037, Telangana, India; 23bhavana11@gmail.com (V.B.); sandeepchary908@gmail.com (P.S.C.)

**Keywords:** antioxidant, anti-inflammatory, nanoemulgel, diabetic wound healing, ferulic acid

## Abstract

**Aim:** To investigate the anti-inflammatory, antioxidant, and diabetic wound healing properties of the novel topical formulation [Ferulic acid-loaded nanoemulgel (DLMGO-G)]. **Methods:** Ferulic acid nanoemulsion developed with lemongrass oil is investigated in diabetic wound healing. Further nanoemulsion is incorporated into 1% carbopol^®^ 934 to obtain the DLMGO-G. Nanoemulsion was characterized for particle size, and polydispersity index (PDI) was obtained by Malvern Zetasizer (Zetasizer Nano ZS, Malvern, AL, USA), and morphology by TEM (JEM 1400, JOEL, Akishima, Japan). Furthermore, in vitro cell line and in vivo studies were carried out. **Results:** The developed nanoemulsion showed a globule size of 28.04 ± 0.23 nm and PDI of 0.07 ± 0.01. The morphology of nanoformulations by TEM confirmed the spherical and uniform nature. Further, the nanoformulation in in vitro cell line experiments revealed that the IC50 value was increased by 1.52 times compared to the drug solution. The treatment groups have shown that fibroblast morphologies were spindle-shaped, suggesting that nanoformulation was compatible with the cells and developed normally on nanoformulation. It also reduced ROS with improved internalization more than the control group. The in vitro wound healing model also revealed that nanoformulation had better wound healing activity. In the in vivo diabetic wound studies on male SD rats, the levels of inflammatory markers such as TNF-α, IL-6, IL-22, and IL-1β declined significantly when treated with DLMGO-G. IL-10 levels significantly increased compared to the diseased group, and MMP-9 levels were remarkably decreased compared to the diseased group. Furthermore, histopathological studies showed the regeneration and granulation of tissues. **Conclusions:** Thus, these findings indicate that FA-loaded nanoemulgel greatly accelerates the healing of wounds in diabetic rats.

## 1. Introduction

Diabetes Mellitus is one of the metabolic conditions with numerous causes and is characterized by chronic hyperglycemia. It can lead to some long-term consequences, such as nephropathy [1], renal failure, neuropathy [2], and progressive development of retinopathy with an inherent risk of blindness [3]. These long-term consequences may result in Charcot joints, foot ulcers, amputations, and autonomic dysfunction, which may include sexual dysfunction [4]. Poor wound healing is a generally long-term consequence of diabetes mellitus, resulting in approximately 50% of non-traumatic amputations. Diabetes increases the risk of wound complications, including open, non-healing ulcerations of the foot, leg, or toes. A study found that at least 15% of diabetes patients develop non-healing wounds at a certain time. In 81% of cases, poor wound healing was the primary contributing factor, according to a recent retrospective study that explored the root causes of multiple cases of diabetic amputations [5].

Patients with diabetes have impaired angiogenesis and neovascularization, which negatively impacts the wound-healing process. Moreover, diabetes individuals are more vulnerable to infections and ulcers that can lead to gangrene due to impairments in the functions of matrix metalloproteinase (MMPs), keratinocytes, and fibroblasts [6].

Hyperglycaemia leads to increased generation of superoxide anion and reactive oxygen species (ROS) in the mitochondrial electron transport chain [7]. ROS is a key player in the pathophysiology of cell malfunction and the challenges associated with diabetes. Additional factors that can impede the healing of wounds include infection, inadequate blood flow, radiation exposure, age, temperature, diet, systemic infection, glucocorticoid medication, and genetic defects such as Danlos syndrome [8]. In this research investigation, our main aim was to target both lowering blood sugar and promoting wound healing at the same time.

Lemon grass oil (LGO) is one of the essential oils that is rich in terpenoids like citral and limonene and other main components, such as geranial, neral, and carvone. The main pharmacological activities are antioxidant [9,10], anti-inflammatory [11], and antimicrobial [12]. Natural antioxidant and phenolic compound ferulic acid (FA; 4-hydroxy-3-methoxycinnamic acid) has a wide range of pharmacological actions, such as anti-inflammatory [13], radioprotective [14], anti-diabetic [15], anti-cancer [14], antioxidant [15], angiogenic [16], antibacterial [17], and neurogenic properties [18]; ferulic acid may show potential synergistic activity in diabetic wound healing. According to earlier research, ferulic acid may elevate platelet-derived growth factor, vascular endothelial growth factor, and hypoxia-inducible factor-1α, which are necessary for cell regeneration and total wound healing [16]. Furthermore, FA efficiently suppressed lipid peroxidation and nitric oxide levels and increased catalase, superoxide dismutase, and glutathione, all of which likely aided wound healing. Due to its hydrophobicity, it has poor aqueous solubility [19,20]. To overcome this obstacle in this present investigation, we made FA-loaded nanoemulgel for wound healing [21].

Nanoemulgel is one of the best delivery systems for hydrophobic molecules due to its better physical stability, non-toxicity, and non-irritating nature [22,23]. The nanoemulgel possesses the hydrogel and nanoemulsion attributed to the controlled drug release, and nano-size would help permeate the release components along with the bacterial and fungal activities [21,24,25].

Recently, our lab published results on the FA-loaded nanoemulgel, which showed a better therapeutic response in wound healing only [21] with a droplet size of 26.15 ± 0.58 nm. The nanoformulation of drug content was found to be 98.11 ± 0.51%. The developed FA-loaded nanoemulgel (DLMGO-G) showed pseudoplastic behavior. The DPPH free radical percentage inhibition was 52.82 ± 0.33% and 75.62 ± 0.16% for blank lemongrass oil nanoemulsion loaded gel (LGO-G) and DLMGO-G formulation. The formulations also showed in vitro antibacterial activity against *S. aureus*. The reduction in inflammatory mediators, such as IL-6 and TNF-α levels, was observed to a greater extent in BLMGO-G and DLMGO-G when compared to the negative group. The research findings are based on the topical delivery of FA from optimized DLMGO-G followed by the enhancement of in vitro antioxidant activity [26], antibacterial activity [26], and in vivo wound healing effects in rat excision wound models [21].

In this present research investigation, our main aim is to explore the topical formulation for diabetic wound healing [21] in comparison with the marketed formulation in rats with excision wounds.

## 2. Materials and Methods

The ferulic acid was purchased from Sigma-Aldrich in St. Louis, Missouri, in the United States. The essential oils (lavender and lemongrass) were purchased from Botanical Healthcare, located in Bangalore, India. The surfactants, which included Tween^®^ 20, Tween^®^ 80, and Carbopol^®^ 934, were acquired from SRL Chemicals in India, while a gift sample of Cremophor^®^ELP was received from BASF in India. The additional surfactants, such as Labrasol^®^ and Transcutol^®^P, were purchased from Gattefosse, India. Solvents of HPLC analytical grade, such as methanol and acetonitrile, were purchased from SRL Chemicals in India.

### 2.1. Formulation and Characterization of Nanoemulsion

Nanoemulsion was designed, characterized, and fabricated using a low-energy technique; an emulsion inversion point was used as per our author’s laboratory work [21]. The previously described procedure was used to create the Fan A-loaded LMGO-based nanoemulsion. The low energy method of emulsion inversion point was used to prepare the FA-loaded LMGO-based nanoemulsion. FA (1%) was added to the beaker containing an oil and surfactant mixture of Transcutol^®^P and Cremophor^®^ELP (1:1) while being continuously stirred by a magnet [20]. Finally, the combination was microtitrated with water and designated DLMGO with FA and BLMGO without FA.

The particle size of the nanoemulsion was determined using the dynamic light scattering (DLS) method. To prepare the sample, the nanoemulsion was diluted 10 times with deionized water. Using a zetasizer, we loaded the sample cell with nanoemulsion (Zetasizer Nano ZS, Malvern, AL, USA), and following the proper standard operating procedure (SOP), the globule size was determined. The cell temperature was kept constant at 25 °C during the three measurements that were conducted [27]. The FA-loaded nanoemulgel was designed, characterized, and fabricated as per our author’s laboratory-published work on wound healing [21]. The optimized nanoemulgel was characterized morphologically using transmission electron microscopy (TEM). For the TEM study, the diluted FA-loaded nanoemulsion (DLMGO) was immobilized on carbon-coated copper grids. To improve the imaging, two percent of uranyl acetate was used to stain the diluted DLMGO-GEL. A CCD CAMERA EMSIS camera was used to capture the images (JEM 1400, JOEL, Akishima, Japan).

### 2.2. In Vitro Cell Line Studies

The mouse fibroblast cell line (L929) was purchased from NCCS (National Center for Cell Science), Pune, India. Cells were cultured in DMEM media, including 10% FBS; 1% penicillin–streptomycin antibiotic was used.

#### 2.2.1. MTT Assay

To check the cytotoxicity of FA and nanoformulation MTT assay was performed. In this assay, mouse fibroblast (L929) cell lines were seeded in a 96-well plate, including DMEM media, 10% FBS, incubated for 24 h at 37 °C, and 5% CO_2_ incubator. After the incubation, the media was removed, and fresh media was added; then, the cells were treated (FA and nanoformulation) and incubated for 24 h. Later, 100 μL of MTT reagent was added to each well and incubated for four hours. After 4 h, the MTT reagent solution was aspirated, and then, 100 μL of DMSO was added to dissolve the formazan crystals. The absorbance of a 96-well plate was taken using a multimode reader (Synergy H1, BioTek Instruments, Winooski, VT, USA); the IC50 (inhibitory concentration) values were calculated [28].

#### 2.2.2. Cellular Uptake Study

A cellular uptake study was performed to reveal the uptake of nanoformulation-loaded FITC fluorescent dye in L929 cells. The cells were plated in a 12-well plate and grown in DMEM, 10% FBS, and incubated for 24 h. Later, dye-loaded nanoformulation was added to the plate and incubated for 6 h at 37 °C in a 5% CO_2_ incubator. Later, fluorescent imaging was performed using the Nikon Ti2 microscope (Tokyo, Japan).

#### 2.2.3. 2,7-Dichlorodihydrofluorescein (DCFDA) Staining

Production of ROS (Reactive oxygen species) in cells can be detected by DCFDA (2,7-dichlorofluorescein diacetate). This assay shows the oxidative conservation of stable nonfluorescent 2,7-dichlorodihydrofluorescein (DCFDA) to highly fluorescent 2,7-dicholorofluorescin (DCF). The conversion happens when DCFDA enters the cells, interacts with ROS, and produces green fluorescence. The L929 cells were plated in a 12-well plate, supplemented with DMEM and 10%FBS, and incubated for 24 h. After 24 h of incubation, the cells were exposed to LPS with IC50 value and a high dose of nanoformulation and incubated for 24 h. After 24 h of incubation, the 12-well plate was treated with DCFDA for 30 min, and images were captured using a fluorescent microscope (Nikon Eclipse TiS, Tokyo, Japan) at 20× magnification.

#### 2.2.4. Acridine Orange and Ethidium Bromide (AO/EB) Dual Staining

AO/EB dual staining is one of the methods to evaluate cell apoptosis. Apoptosis is one of the main mechanisms that alter the angiogenesis and wound-healing process [29]. In this study, the L929 cells were grown in a 12-well plate and incubated overnight. After incubation, the cells were exposed to LPS with IC50 value and a high dose of nanoformulation and incubated for 24 h. The next day, we changed the media and added fresh media. Later, ten microliters of dye solution, which consisted of acridine orange and ethidium bromide, was added. Live cells were stained with acridine orange, and dead cells were stained with ethidium bromide.

#### 2.2.5. Morphology Study

In order to investigate the morphology of L929 cells, cells were seeded in a 12-well plate and incubated for 24 h. After incubation, cells were treated with LPS, IC50, and a high dose of nanoformulation and incubated for 24 h. The morphological changes, such as size and shape, were observed using an inverted microscope (Nikon Eclipse TiS, Tokyo, Japan).

#### 2.2.6. Scratch Assay

To evaluate the potential role of nanoformulation in wound closure, an in vitro wound model was developed in L929 cell lines. In a 6-well plate, L929 cells were seeded, with 1 × 10^6^ cells per well. After 90% of confluence, we made a gentle scratch on the monolayer to create a wound, and PBS was used to wash the cells to discard the dead cells. Then, cells were exposed to LPS with IC50 and a high dose of nanoformulation and incubated for 24 h. Images were taken at the 0th hour and 24 h using an inverted microscope (Nikon Eclipse TiS, Tokyo, Japan). We used Image-J software version 1.54 (NIH, Bethesda, MD, USA) to measure the wound area in order to quantify the data.

### 2.3. Evaluation of Wound Healing Processes Employing a Rat Excision Wound Model

In order to assess the nanoemulgel formulation’s therapeutic efficacy as a therapeutic agent, in vivo studies were conducted on male Sprague Dawley (SD) rats weighing between 250 and 300 g. We procured rats from Vyas Laboratories Pvt. Ltd. Hyderabad, India. The rats were acclimatized before the start of this experiment. The protocol was approved by the Institutional Animal Ethics Committee and NIPER Hyderabad, India (Protocol no. NIP/12/2021/PE/435). The in vivo studies were carried out while closely adhering to the guidelines laid by the Committee for Control and Supervision of Tests on Animals (CCSEA). All experimental procedures were reported in accordance with the ARRIVE guidelines specifications [30].

**Figure 1:**
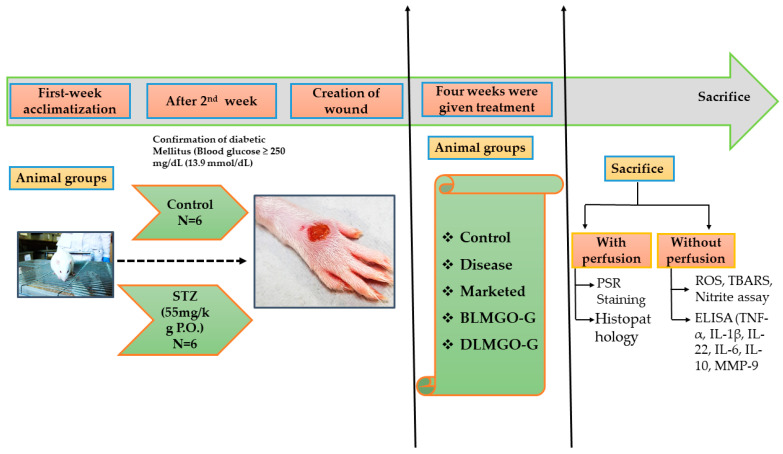
The schematic representation of the in vivo study design to assess the formulation on STZ-induced SD rats.

In the present study, rats weighing 250–300 g were used. The wound healing area was assessed in the following groups: Group I (subjects with wounds but no treatment in the healthy group); Group II (subjects in the disease group who had a streptozocin (STZ) wound but were not receiving treatment); Group III (subjects treated with the Povidone iodine [marketed ointment] + STZ); Group IV (treated with STZ + BLMGO-GEL), and Group V (treated with STZ + DLMGO-GEL), as shown in Figure 1. The grouping and number of each group of animals are described in Table 1.

To develop diabetes, rats received an intraperitoneal (IP) injection of 45–55 mg/kg streptozocin (in 0.01 M sodium citrate; pH 4.3). Every day, blood was collected from the tail vein, and the blood glucose (BG) level was measured using a glucometer. If blood glucose levels were more than 16.7 mmol/L, rats were selected as diabetic and maintained within 16.7 to 33.3 mmol/L with 6–18 units of insulin per day [31]. After two weeks, rats were exposed to isoflurane for anesthesia to develop the diabetic wound, which was created with the help of a disposable 5 mm skin biopsy punch and Westcott scissors on the dorsal hind foot of the diabetic rats.

Every rat in each group was given the developed nanoemulgel topically twice a day for up to 21 days. The healing period of wounds was measured to evaluate the wound healing, as shown by Equation (1). We visually determined the wound area on alternate days using a scale. ImageJ software, version 1.54 was used to measure the closing area and the overall wound healing area. On the last day, blood glucose levels were checked, and the animals were sacrificed by inhalation anesthesia (isoflurane); blood was collected from retro-orbital using anti-coagulant heparin vials for estimation of blood glucose levels, and their skins were collected. Upon sectioning the skin samples, a portion was immersed in a 10 v/v% formalin solution for further histological examination, and a small number of samples were kept at −80 °C in order to conduct additional biochemical tests such as oxidative stress parameters and inflammatory and anti-inflammatory markers estimated by ELISA; MMP-9 levels were also estimated by ELISA.

(1) $$Wound\;contraction\left(\%\right)=\frac{\left(A_{0}-A_{n}\right)}{A_{0}}\times 100$$

where A_0_ is the first day of wound area; A_n_ is the nth day of wound area.

#### 2.3.1. Glucose Oxidase (GOD) Peroxidase (POD) Assay

In 1.5 mL heparinized centrifuge tubes, blood was collected from the rat’s retro-orbital to separate plasma by centrifuging the mixture at 5000 rpm for five minutes. Plasma glucose was estimated using the GOD-POD assay kit (Accurex, Mumbai, India), and the data were indicated in mg/dL, as per the manufacturer’s instructions [32].

#### 2.3.2. Oxidative Stress Parameters

##### Nitrite Level

As per the protocol reported by Urati, Anganti et al. (2023) [33] was used to measure the nitrite content in the skin homogenates. In a 96-well plate, an equal quantity of Griess reagent and skin tissue supernatant were combined and incubated for 10 min in the dark, and the nitrite content was determined by measuring the absorbance at 540 nm. With sodium nitrite serving as the standard, the nitrite levels were determined using a standard curve, and the protein levels were shown in μmol/mg.

##### Thiobarbituric Acid Reactive Substances

As per the protocol by Ohkawa, Ohishi et al. (1979), the lipid peroxidation in the skin tissue was estimated using the thiobarbituric acid-reactive substance (TBARS) assay [34,35,36]. The skin tissue samples were mixed thoroughly in cooled phosphate buffer. In the meantime, the tubes with 8.1% SDS, 20% acetic acid, and 0.8% TBA were filled with the tissue homogenate. The reaction mixture was cooled with running tap water following one hour of incubation at 95 °C. Later, the samples were centrifuged at 10,000 rpm for ten minutes. After that, the reading was taken at 532 nm. Micromoles malondialdehyde (MDA) per mg protein are the results of calculating the MDA level using 1,1,3,3-tetra methoxy propane (97%), based on the standard curve.

#### 2.3.3. Enzyme-Linked Immunosorbent (ELISA) Study of Pro- and Anti-Inflammatory Cytokines

Cytokine concentration increases with inflammation, making it one of the most important variables to consider while assessing a state of inflammation. The ELISA test was used to measure the important biomarkers essential for the treatment. In all groups, the levels of pro-inflammatory cytokines such as IL-1β, IL-22, IL-6, and TNF-α, as well as anti-inflammatory cytokines like IL-10, were measured. With the aid of a homogenizer (Remi Electrokinetic, Ltd., Mumbai, India), the tissue samples were homogenized using lysis buffer for five minutes at 3000 rpm. The homogenates were centrifuged for ten minutes at 4 °C at 10,000 ± 100 rpm. Additionally, the supernatant was separated and kept at −80 °C. The stored samples were cooled to room temperature, following the instructions given by the manufacturer, and then the levels of cytokines were measured using ELISA kits (R&D Systems, Inc., Minneapolis, MN, USA). The data were expressed in pictograms per milligram protein after the interleukin levels were normalized using the Bradford protein assay.

#### 2.3.4. Estimation of Metalloproteinase-9 (MMP-9) Levels

The determination of metalloproteinase-9 (MMP-9) was performed in the plasma samples using a commercially available kit (R&D system, Minneapolis, MN, USA; Catalogue number: R&D DY8174). In summary, 100 μL of capture antibody was added to a 96-well ELISA plate, and it was left to incubate at room temperature overnight. The wells were subsequently rinsed three times using a wash buffer that contained 0.05% Tween 20 in PBS 0.01 M (pH 7.4). Following the addition of blocking buffer (1% BSA; 300 µL) to the plate, test samples or standard (100 μL) samples were added and allowed to incubate for 2 h at RT. After the addition of 100 μL of the diluted detection antibody to each well, the wells were incubated for an hour. Next, the plate was washed, and each well received an equal amount of substrate before being incubated for half an hour. In the end, 50 μL of 2N H_2_SO_4_ stop solution was added to each well, and the plate was gently shaken to achieve an equal mixing. At 450 nm, the well’s absorbance was measured immediately. MMP-9 levels were shown as pictograms per milligram.

#### 2.3.5. Histopathological Studies

After embedding the granulation tissue/healing specimens in paraffin wax and fixing them in 10% neutral buffer formalin, 5 µm thick tissue slices were cut and stained with H&E using standard techniques. Using a light microscope, the stained slices were examined. An expert pathologist who was blind to the experimental groups scored the histological characteristics of granulation/healing tissue sections.

Sections were stained with picrosirius red (Direct Red 80 Sigma-Aldrich, USA) using a modified picrosirius method [37,38], and under polarised light, stained sections were analyzed to determine the content of collagen and the thickness of the fibers in the healing wounds. Collagen fibers were distinguished as (i) thick and denser collagen, exhibiting orange-to-red color, based on the birefringence pattern, and (ii) collagen fibers that were thinner and appeared green-to-yellow [39].

#### 2.3.6. Statistical Analysis

GraphPad Prism 8.0.2 (GraphPad Software, Inc., La Jolla, CA, USA) was used to plot the data and perform statistical analysis. One-way analysis of variance was used to analyze the data, and all values were analyzed by one-way ANOVA, followed by Tukey’s multiple comparison test, and expressed as mean ± S.E.M. *p*-values less than 0.05 were regarded as statistically significant. The data have been reported as mean ± standard error mean (SEM) of the triplicate (*n* = 3) independent studies.

## 3. Results and Discussion

### 3.1. Droplet Size and PDI

**Figure 2:**
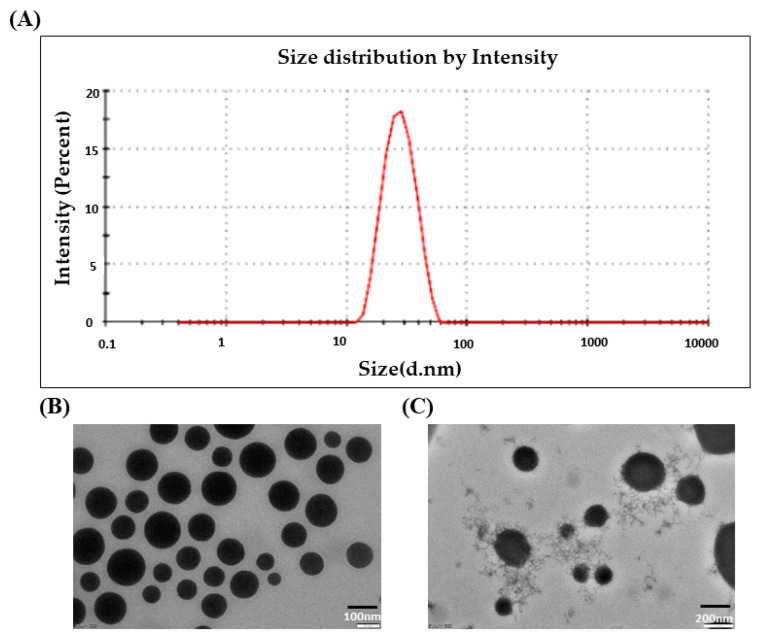
Characterization of drug-loaded nanoemulsion (DLMGONE) and drug-loaded nanoemulgel (DLMGO-G); (**A**) Droplet-size distribution analysis of DLMGONE. (**B**,**C**) Transmission electron microscopy (TEM) image of DLMGONE and DLMGO-G with scale 100 nm and 200 nm, respectively.

The droplet size of the drug-loaded nanoemulsion (DLMGONE) is shown in Figure 2A. As shown in Figure 2A, the DLGMONE showed a droplet size of 28.04 ± 0.23 nm and 0.07 ± 0.01 of PDI. The droplet size showed that the distribution of droplets in the NE was more uniform and homogeneous. Other studies, such as the selection of nanoemulsion components, pseudo ternary phase diagram, design of expert, and other characteristic properties of nanoemulgel, were already performed in our previous reported work, and in this present study, we used the same optimized formulation for diabetic wound healing because has already been studied for wound healing [21].

### 3.2. Morphological Characterization of DLMGONE

The morphology of optimized DLMGONE was analyzed by TEM. It was found that the nanoemulsion droplet was spherical, homogenous, and uniform, as shown in Figure 2B.

### 3.3. Preparation, Characterization, and Morphology of FA-Loaded Emulgel of DLMGO-GEL

The viscosity modifier employed was Carbopol^®^ 934 polymer. After the addition of the polymer, the DLMGNE was let to soak for 12 h. Triethanolamine was then used to keep the system’s pH stable, which led to the development of DLMGO-G. The 1, 2, and 3% nanoemulgel formulations were developed, and based on their viscosity and anti-gravity effects, the 2% nanoemulgel was chosen for future studies.

The 2% DLMGO-G is whitish–yellow in physical appearance, and the pH is 6.53 ± 0.04 with uniform consistency with the absence of grittiness. It was discovered that the drug content of nanoemulgel (one gram of nanoemulgel weighed and dissolved in organic solvent and incubated for 2 h in a water bath provided with a mechanical shaking option to dissolve FA completely, and the obtained sample was filtered, and FA was quantified with HPLC at 287 n, and the results were found to be 98.11 ± 0.51. Other studies, such as rheology, spreadability, drug assay, in vitro drug release study, drug release kinetics, and ex vivo permeation study, were performed in our previous work [21], showing good results for wound healing. The rheology results of DLMGO-G of different concentrations (1%, 2%, 3%) showed shear thinning and pseudoplastic behavior. The decline in viscosity may be due to the destruction of the gelling matrix on the application of force at a particular point. The 2% DLMGO-G was selected for further studies for the applicability of the sample. The firmness, hardness, and adhesiveness parameters of the nanoemulgel were studied using the force–time graph. DLMGO-G has excellent spreadability and capacity to flow (firmness) on the application of force, as confirmed by the texture analyzer in our previous study. The gel is considered more spreadable when both the firmness and hardness values are low [21]. The antioxidant activity was higher in nanoemulgel (75.62 ± 0.16%) compared to blank nanoemulgel (52.82 ± 0.33%). Therefore, the FA-loaded nanoformulations showed approximately a 17% increase in inhibition of DPPH. The significant antioxidant activity of the formulations was due to FA. In vitro release study revealed that the plain FA gel showed a release of 61.79 ± 4.35% for 30 min, followed by 77.98 ± 4.69% release at the sixth hour. Whereas in DLMGO-G, a release of 34.46 ± 8.00% for 30 min followed by 99.25 ± 3.31% for 6 h was observed. The oil that reserved the FA in the nanoemulgel would be the reason for the controlled release. The results suggest that FA is being released in a controlled way for a period of 6 h [21]. The release of FA from DLMGO-G, followed by permeation through the membrane to receptor media, was demonstrated using isolated rat skin by Franz diffusion apparatus. The permeation of FA from DLMGO-G after 6 h was 25.82 ± 1.42%, and from FA gel, it was 47.98 ± 2.7%. The permeation from DLMGO-G exhibited sustained release up to 12 h. The permeation of FA from DLMGO-G was approximately 22% slower than FA gel due to the encapsulation of FA in nanoemulsion. The findings of the release study concluded that the soluble FA from FA gel could penetrate faster than DLMGO-G [20,21]. So, in the present study, we need to check its activity for diabetic wound healing. Further studies should be carried out for further analysis.

### 3.4. Morphological Characterization of DLMGO-G

The optimized DLMGO-G was characterized morphologically using transmission electron microscopy (TEM), as shown in Figure 2C. For the TEM study, the diluted DLMGO-G was immobilized on carbon-coated copper grids. To improve the imaging, two percent of uranyl acetate was used to stain the diluted DLMGO-G. A CCD CAMERA EMSIS camera was used to capture the images (JEM 1400, JOEL, Japan).

### 3.5. In Vitro Antioxidant and Antimicrobial Assay

Free radicals proliferate in the wound site throughout the healing process, leading to oxidative stress. This damaging condition has been connected to tissue damage, infection, inflammation, and immunosuppression [40]. Therefore, the formulation needs to have the ability to scavenge free radicals. FA functions as an antioxidant by scavenging free radicals and minimizing the damage oxidative stress produces [13]. Early on, bacterial growth may be stimulated by skin disturbance or damage from a wound, leading to wound infection. Studies have demonstrated that FA was responsible for the formulations’ significant antioxidant efficacy, showing higher results in drug-loaded nanoemulgel (75.62 ± 0.16%) compared to blank nanoemulgel (52.82 ± 0.33%). Therefore, the FA-loaded formulations showed approximately a 17% increase in inhibition of DPPH. The significant antioxidant activity of the DLMGO-G was due to FA [21]. There was no significant difference between the NE due to the absence of antibacterial activity of FA against *S.aureus,* clearly indicated by the high MIC value for FA. The significant antibacterial activity of NE might occur due to their interaction with microbial cell membranes. Strong electrostatic interaction with the bacterial cell wall is produced by the vast surface area and passive transport of NE across the cell membrane, which results in antibacterial activity. The fusion of nanoemulsion with the cell membrane and stability of essential oil constituents leads to an increase in the concentration of essential oil at the desired site of action. We had previously discussed this study’s data in our earlier work [21].

### 3.6. Cell Culture

#### 3.6.1. MTT Assay

**Figure 3:**
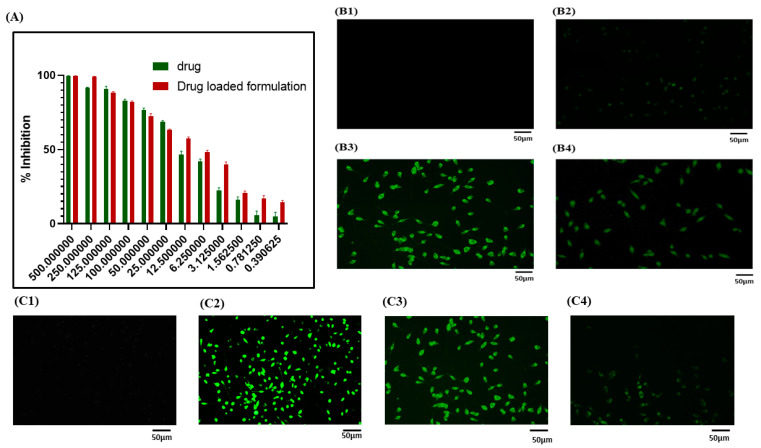
(**A**) Cytotoxicity study by MTT assay of nanoformulation. Cellular uptake study (**B1**–**B4**): (**B1**) Control group; (**B2**) An amount of 10 UL of dye solution; (**B3**) IC50 of dye-loaded nanoformulation; and (**B4**) High dose of dye-loaded nanoformulation. DCFDA staining (**C1**–**C4**): (**C1**) Control group; (**C2**) LPS-treated group; (**C3**) IC50 of nanoformulation; and (**C4**) High dose of nanoformulation.

To evaluate the cytotoxicity of nanoformulation on L929 cells, the MTT reagent was used. The cytotoxicity response of nanoformulation and plain drug solution is shown in Figure 3A, and drug IC50 value was found to be 15.16 μg/mL, whereas 1.52 folds were increased in nanoformulation compared with FA at 24 h.

#### 3.6.2. Cellular Uptake Study

A cellular uptake study was carried out on nanoformulation using fluorescent microscopy (NIKON-Ti2, Melville, NY, USA). In this study, the L929 cell line was employed to check the cellular uptake of dye-loaded nanoformulation. This internalization of cells by nanoformulation was mainly based on the endocytosis mechanism. Figure 3(B1–B4) explains that our nanoformulation can permeate through cells and produce green light.

#### 3.6.3. 2,7-Dichlorodihydrofluorescein (DCFDA) Staining

DCFDA staining was used to evaluate ROS in the L929 cell line; when cells are exposed to LPS, the generation of ROS will occur, which may disrupt the cell membrane and cell organelles [41], which will further cause alteration of wound healing. Figure 3(C1–C4) shows that DCFDA staining decreased in ROS found in nanoformulation.

#### 3.6.4. Acridine Orange and Ethidium Bromide (AO/EB) Staining

**Figure 4:**
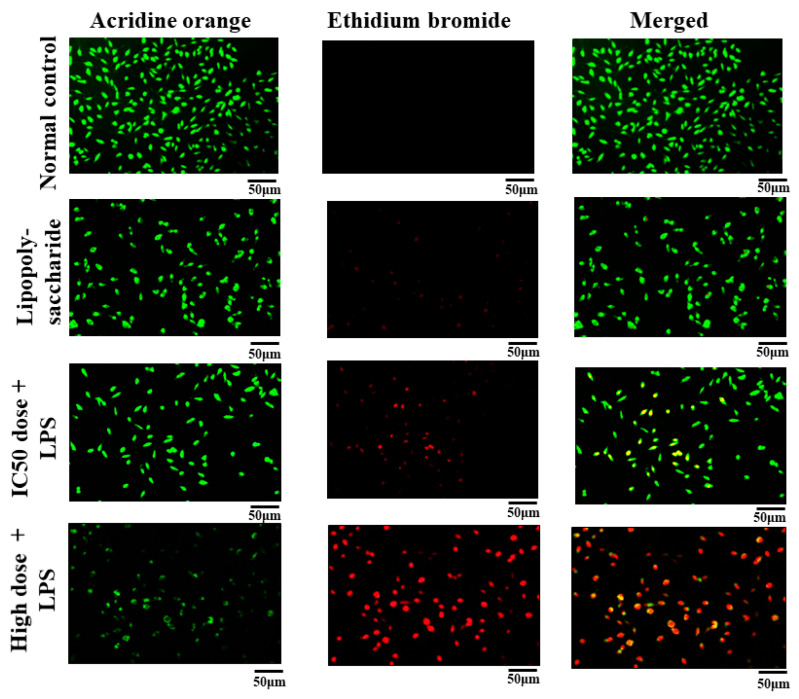
Acridine orange and ethidium bromide (AO/EB) dual staining of nanoformulation.

In the present study, we evaluate apoptosis by utilizing dual staining of AO/EB in L929 cell lines. Necrotic cells were stained by ethidium bromide, whereas early and live apoptotic cells were stained by acridine orange, which were represented as green fluoresces [29]. This study demonstrated that morphological modifications were seen in L929 cells subjected to nanoformulation. There were more apoptotic cells seen. Figure 4 shows the AO/EB staining of L929 cells.

#### 3.6.5. Morphology

**Figure 5:**
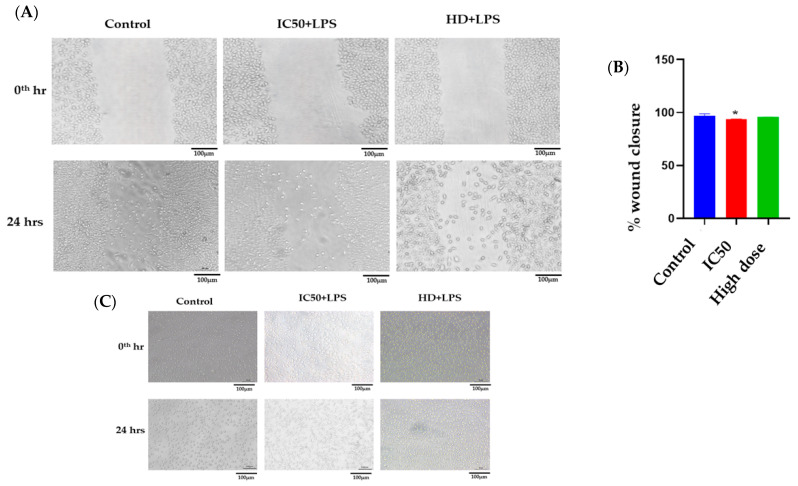
(**A**–**C**): (**A**) In vitro scratch assay of nanoformulation (IC50 and high dose) for wound closure on L929 cell lines. (**B**) % wound closure was quantified using ImageJ software, version 1.45. * *p* < 0.05 vs. Control. (**C**) Morphological assay of nanoformulation.

A morphology assay was performed on L929 cells to check the morphological effects on treatment with nanoformulation. All of the treatment groups have shown that fibroblast morphologies seemed to be spindle-shaped, suggesting that our nanoformulation was compatible with the cells and that the cells developed normally on nanoformulation, as shown in Figure 5C. Additionally, we saw fibroblast proliferation at 24 h, suggesting that nanoformulation supported both the proliferation and viability of fibroblasts.

#### 3.6.6. Scratch Assay

Fibroblast cells were important for wound healing because they helped in the development of granulation tissue, extracellular matrix, and collagen. Migration is a normal process that helps in wound closure after tissue damage. L929 cells were used to create an in vitro wound healing model [42,43,44]. After an injury to the monolayer of cells, treated groups showed wound closure. IC50 of nanoformulation was significantly (* *p* < 0.05) improved compared to the control group, whereas, in the high-dose group, IC50 of nanoformulation improved non-significantly compared to the control group, as shown in Figure 5A,B.

### 3.7. Evaluation of Wound Healing Processes Employing a Rat Excision Wound Model

**Figure 6:**
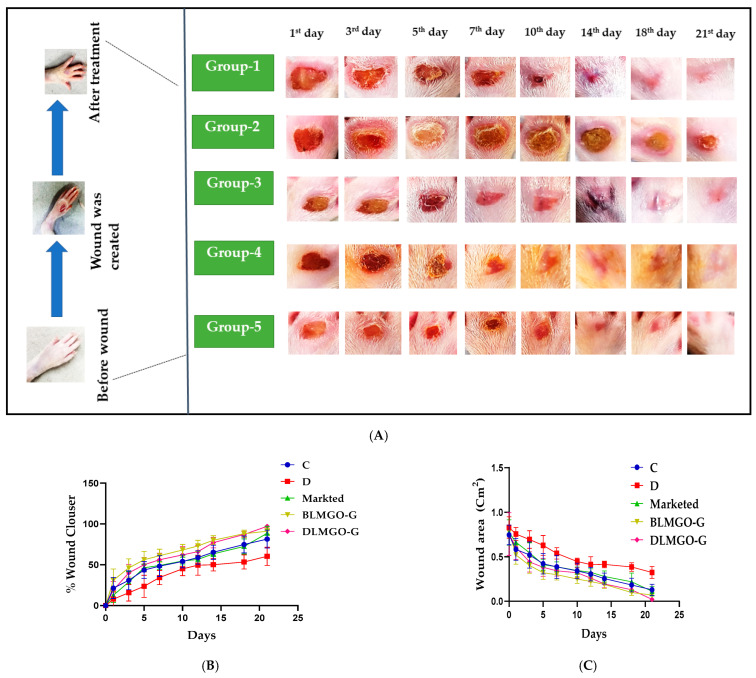
(**A**–**C**): (A) Dorsal view phenotypic photos exhibiting the subjects; (**B**) The % of wound closure of each group on different days; (**C**) The wound area of each group on different days.

The BLMGO-G and DLMGO-G marketed formulations were tested for their effects on diabetic wound healing using an animal model of foot ulcer. For 21 days, the animals received the treatment, and the wound area was recorded on an alternate day. On the 21st day following the diabetic wounds, the animals were sacrificed for histological and biochemical analyses. After the seventh day, the marketed formulation and DLMGO-G revealed faster wound healing than the STZ group, and the blank gels also showed faster wound healing. On day 18 after wounding, a nearly 87 ± 3.83% closure was revealed for drug-loaded gels. On day 21, it was over 97 ± 2.11% (Figure 6B,C), in comparison to the STZ group; significantly improved wound area and wound closure were found in the treatment group. The dorsal view of phenotypic images is shown in Figure 6A.

#### 3.7.1. Effect of Body Weight and Blood Glucose Level

**Figure 7:**
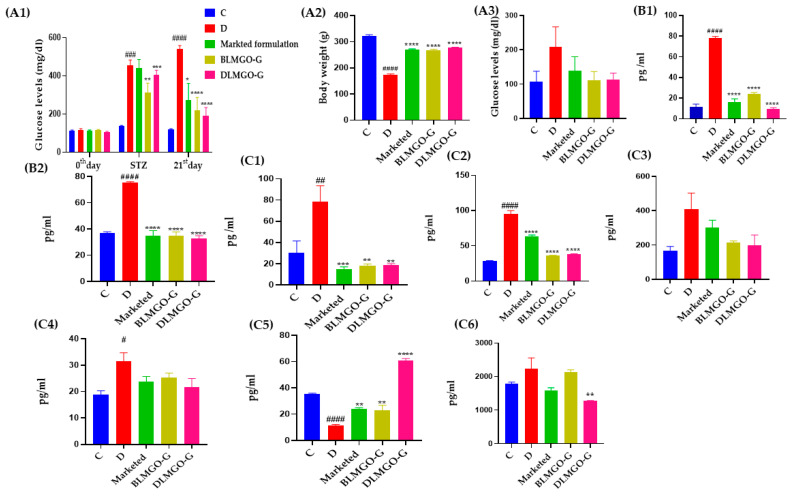
(**A1**–**C6**): (**A1**) Glucose levels by glucometer of different groups on different days; (**A2**) Bodyweight of each group on different days; (**A3**) God-pod assay of different groups; (**B1**,**B2**) Effect of Nitrite and TBARS levels on STZ intoxication and Novel nanoformulation treatment; (**C1**–**C5**) Effect of inflammatory and anti-inflammatory cytokines such as TNF-α, IL-1β, IL-22, IL-6, and IL-10 on STZ intoxication and Novel nanoformulation treatment; (**C6**) MMP-9 levels of different groups. All values were analyzed by one-way ANOVA followed by Tukey’s multiple comparison test and expressed as mean ± S.E.M and ^####^ *p* < 0.0001 vs. Control (C), ^###^ *p* < 0.001 vs. C, ^##^ *p* < 0.01 vs. C, ^#^ *p* < 0.05 vs. C, **** *p* < 0.0001 vs. disease (D), *** *p* < 0.001 vs. D, ** *p* < 0.01 vs. D, * *p* < 0.05 vs. D.

After induction of STZ in all groups, changes in body weight levels were observed except for the control group. Then, after 21 days of treatment in all groups except the control group, the diseased group’s body weight was found to have significantly decreased. Other treated groups had normal body weight, similar to the control group. Before induction of STZ, glucose levels were checked with a glucometer (Dr. Morepen BG-03 Gluco One, Glucometer purchased from Apollo pharmacy, Hyderabad, India). After induction of STZ, glucose levels were measured and monitored. If glucose levels were more than 250 mg/dL, it indicated that diabetes was confirmed, and later, a diabetic wound was created. If glucose levels were more than 350 mg/dL, then insulin was given to maintain glucose levels. Before sacrificing on the 21st day, glucose levels were checked again and were found to be non-significantly decreased compared to the STZ induction data shown in Figure 7(A1). After sacrifice, blood was collected from retro-orbital, and plasma was isolated from that blood and further analyzed to check the glucose levels by GOD-POD assay; it was found that the STZ-treated group had non-significantly increased glucose levels compared to the control group, whereas DLMGO-G and BLMGO-G groups had insignificantly decreased glucose levels compared to the diseased group, as shown in Figure 7(A3).

#### 3.7.2. Oxidative Stress Parameters

Few studies showed that administration of FA systemically and/or topically decreased inflammation and oxidative stress and expedited the rate of wound healing or re-epithelialization [45,46]. We also observed the change in skin nitrite levels and MDA levels, as shown in Figure 7(B1,B2). In the STZ-treated group, it was found that skin nitrite levels and MDA levels were significantly enhanced. Whereas in treatment groups, they found significantly decreased compared to the STZ-treated group.

#### 3.7.3. Inflammatory and Anti-Inflammatory Markers

During the phases of wound healing that involve coagulation, inflammation, proliferation, and remodeling, a variety of cytokines and growth factors, including interleukin IL-1β, IL-22, IL-6, and tumor necrosis factor TNF-α, are elevated [47,48]. Additionally, levels of anti-inflammatory mediators, such as IL-10, decrease [49,50]. Vascular endothelial cells, keratinocytes, and fibroblasts quickly release TNF-α in the damaged area, which triggers the inflammatory phase by attracting inflammatory leukocytes.

During the inflammatory reaction, major sources of TNF-α are recruited by neutrophils and macrophages, triggering a positive acceleration cycle that prolongs inflammatory responses. The proliferation and remodeling stages of wound healing are significantly and directly influenced by interleukins such as IL-1β, IL-22, and IL-6, which stimulate the synthesis of collagen and angiogenesis [51].

An alteration in skin inflammatory cytokines (that is, TNF-α, IL-1β, IL-22, and IL-6) is illustrated in Figure 7(C1–C4). When compared to the control group of rats, there was a substantial change in skin inflammatory cytokines such as TNF-α, IL-1β, IL-22, and IL-6 after STZ exposure. It was observed that in the STZ-treated group, inflammatory cytokines TNF-α, IL-1β, IL-22, and IL-6 dramatically increased. In contrast, treatment groups observed a significant reduction when compared to the STZ-treated group. The change in anti-inflammatory cytokines such as IL-10 is shown in Figure 7(C5). In the STZ-treated group, a decreased level of anti-inflammatory cytokines, such as IL-10, was found, whereas, in the treatment group (DLMGO-G), it significantly increased compared to the STZ group.

#### 3.7.4. Estimation of MMP-9 Levels by ELISA Kit

The extracellular matrix (ECM) is a key ambient component that facilitates the healing of diabetic wounds by acting as a connecting scaffold for cells to promote tissue growth and regeneration [52]. ECM is quickly formed and deposited by fibroblasts, which is necessary for effective cellular adherence because it provides the structural framework for angiogenesis, the process that regenerates and expands blood vessels [39]. Matrix metalloproteinases (MMPs) are a class of zinc-dependent endopeptidases that are essential for the integrity of the extracellular matrix (ECM). MMPs are produced at a higher rate in diabetic wounds with prolonged inflammation. In non-healing wounds, elevated levels of MMP-1, MMP-8, and MMP-9 have been found [53]. The change in plasma samples of MMP-9 levels is shown in Figure 7(C6). In the STZ-treated group, MMP-9 levels increased insignificantly, as shown in Figure 7(C6), whereas, in the treatment group (DLMGO-G), they significantly decreased compared to the STZ group.

#### 3.7.5. Histopathological Studies

**Figure 8:**
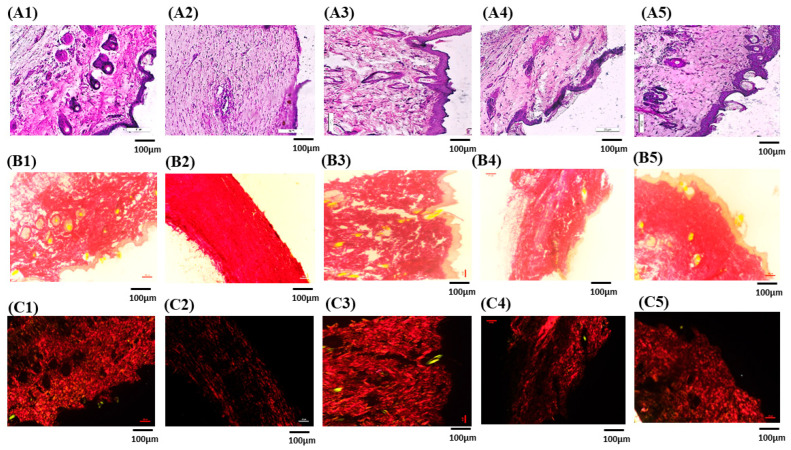
Rat skin stained with hematoxylin and eosin for histopathological analysis (**A1**–**A5**): (**A1**) Healthy group; (**A2**) Disease group; (**A3**) Marketed group; (**A4**) Blank nanoemulgel (BLMGO-G); and (**A5**) Drug loaded nanoemulgel (DLMGO-G). PSR Red staining of rat skin (**B1**–**B5**): (**B1**) Healthy group; (**B2**) Disease group; (**B3**) Marketed group; (**B4**) BLMGO-G; and (**B5**) DLMGO-G. PSR staining of rat skin in polarized light images (**C1**–**C5**): (**C1**) Healthy group; (**C2**) Disease group; (**C3**) Marketed group; (**C4**) BLMGO-G; and (**C5**) DLMGO-G. Each of the histopathology studies has a scale of 100 µm.

To find out how formulations affected the healing process of wounds, histopathological analyses were conducted on the collected skin samples. Images of histological investigations are shown in Figure 8(A1–A5). Skin sections were stained with hematoxylin and eosin (H&E) before being viewed under a microscope. Figure 8(A1–A5) shows the evaluated histopathological wound healing tissue images and, for reference, healthy group, diseased group, BLMGO-G, and DLMGO-G. In the positive and investigative formulations, the normal epidermis with well-healed skin structures and indications of skin remodeling were observed. Rats treated with nanoemulgel (DLMGO-G) showed little to no mononuclear inflammatory cells and an abundance of granulation tissue, along with no evidence of ulcers or edema.

#### 3.7.6. Picro-Sirius Red Staining for Collagen

PSR was used to identify the different forms of collagen present in the extracellular matrix of wound-healing tissues. Under polarized light microscopy, type III collagen was distinguished by greenish birefringence, whereas mature type I collagen fibers displayed yellow and red birefringence [54]. The skin tissue that was stained with picro-sirius and shown in Figure 8(B1–B5, C1–C5) displayed yellowish–red birefringence, which suggested that collagen type I was densely present. Compared to the disease and marketed groups Figure 8(C2,C3), the group treated with DLMGO-G showed a dense network of collagen type I (Figure 8(C4)). Type I collagen fibers were arranged parallel to the skin in both the repaired and normal skin.

## 4. Conclusions

The current investigation provides a spotlight on possible characteristics of ferulic acid nanoemulgel for topical delivery. The phase inversion composition technique was used for the preparation of nanoemulsion, and later, it was converted to gel by carbopol^®^934. In vitro studies on cell lines demonstrated that the IC50 value of the nanoformulation was 1.52 times higher than that of the drug solution, while internalization of the cells had greater cellular uptake of the nanoformulation in comparison to the control group. The production of reactive oxygen species (ROS) was identified using DCFDA staining, and the qualitative apoptosis staining method showed better results. Furthermore, the in vivo investigations showed that topical FA nanoemulgel was useful in accelerating the healing of wounds in diabetic rats. A significant decrease in inflammatory cytokines and MMP-9 levels was shown in the DLMGO-G-treated group, whereas anti-inflammatory cytokine showed significantly elevated levels in the treated group. In this study, the anti-inflammatory, antioxidant, and oxidative stress parameters were evaluated. Significant results against diabetic wounds were noted, thus confirming the promotion of wound healing. The future perspective of this study involves additional molecular research and the need for effective, clinically usable therapy choices, which is the primary barrier to managing chronic wounds. Similar issues, such as a short residence time at the application site, may arise with emulgel and other gel-based formulations. Conversely, a nanoemulgel formulation that must be embedded in the afflicted area may release the drug more slowly and aid in retention for a longer amount of time, which speeds up wound healing.

In summary, the current study heralds an era of innovation in the pharmaceutical profession for exploiting the potential benefits of ferulic acid as one of many therapeutic remedies by extrapolating the fascinating discoveries of ferulic acid-loaded nanoemulgel.

## Figures and Tables

**Table 1:** Description of grouping of animals.

Groups	Treatment	No. of Animals *
Group 1	Control	06
Group 2	Disease: Streptozocin (STZ: 55 mg/kg: i.p.)	06
Group 3	Marketed formulation (Povidone iodine) + STZ	12
Group 4	Blank nanoemulgel (BLMGO-GEL) + STZ	12
Group 5	Drug (FA) loaded nanoemulgel (DLMGO-GEL) + STZ	12
Total		58 *
Considering 10% expected mortality Where * Is total number of animals, i.p. is intraperitoneal route of administration		

## Data Availability

Data will be available upon request.

## List of Abbreviations

DLMGNE	Drug-loaded nanoemulsion
DLMGO-G	Drug-loaded nanoemulgel
BLMGO-G	Blank nanoemulgel
FA	Ferulic acid
MMPs	Metalloproteinase
ROS	Reactive oxygen species
LGO	Lemon grass oil
DLS	Dynamic light scattering
TEM	Transmission electron microscopy
TNF-α	Tumor necrosis factor-alpha
IL-1β	Interleukin-beta
IL-6	Interleukin-6
IL-10	Interleukin-10
IL-22	Interleukin-22
MDA	Malondialdehyde
TBARS	Thiobarbituric acid-reactive substance
MMP-9	Metalloproteinase-9
ELISA	Enzyme-linked immunosorbent
MIC	Minimum inhibitory concentration
STZ	Streptozocin
BG	Blood glucose
GOD	Glucose oxidase
POD	Peroxidase
